# The theory of a co‐creative process in advanced palliative home care nursing encounters: A qualitative deductive approach over time

**DOI:** 10.1002/nop2.203

**Published:** 2018-10-08

**Authors:** Elisabeth Bergdahl, Britt‐Marie Ternestedt, Carina Berterö, Birgitta Andershed

**Affiliations:** ^1^ Faculty of Nursing and Health Sciences Nord University Bodø Norway; ^2^ Department of Health Care Science/Palliative Research Centre Ersta Sköndal Bräcke University College Stockholm Sweden; ^3^ Division of Nursing Science, Department of Medical and Health Sciences Linköping University Linköping Sweden; ^4^ Faculty of Health, Care and Nursing Norwegian University of Science and Technology Gjøvik Norway

**Keywords:** caring, case study research, nurse–patient relationship, palliative care

## Abstract

**Aims and objectives:**

The aim of this study was to test the theoretical conceptualization of the co‐creative process in home care nursing encounters over time.

**Method and design:**

This was a multiple case study with a deductive analysis of qualitative data over time, using interviews and observations collected from three cases.

**Results:**

The co‐creative process was complex and contained main, sub‐ and micro‐processes. Time was important and valuable, giving the patient and relatives space to adjust the process to their own pace. Some processes were worked on more intensively in accordance with the patients’ and relatives’ needs, and these are considered the main‐process. The further developed theory of the co‐creative process and its main, sub‐ and microprocesses can be understood as a concretization of how good nursing care can be performed within caring relationships in the context of advanced palliative home care.

## 1 INTRODUCTION

The aim of palliative care is to give the best possible quality of life for patients and their families. Palliative care represents a philosophy of care that should be an integral part of care and should take place in any setting; it is not necessarily connected with specific institutions. Palliative care should also enable patients to die with dignity while also helping their families during bereavement (WHO, 2016).

### 1.1 Background

Palliative care remains a complex area of clinical practice (Barnard, Hollingum, & Hartfiel, 2006; Broom, Kirby, Good, & Lwin, 2015). Nurses working with palliative care, no matter the context, are expected to deliver a high quality of care, including but not limited to pain treatment, symptom control and general care. The nurse is also expected to create trustful relationships and to give counselling to the patient and the family. The most important consideration for patients in this context is “living a meaningful life,” which is highlighted in a systematic mixed studies review by Sandsdalen, Hov, Høye, Rystedt and Wilde‐Larsson (2015). Responsive healthcare personnel that give the patients an opportunity for participation in the care are important when the patients are striving for what they regard as meaningful in this phase of life.

Healthcare providers should also promote caring relationships and communication by working as a team with patients and relatives in a co‐creative process, where the patients’ needs and quality of life are in focus (Barnard et al., 2006; Bergdahl, Benzein, Ternestedt, Elmberger, & Andershed, 2013; Broom et al., 2015). However, the nurses’ task of creating caring relations has been described as a challenge (Stajduhar, Funk, Roberts, Cloutier‐Fisher, et al., 2011; Stajduhar, Funk, Roberts, McLeod, et al., 2011). In a metasynthesis, Lindahl Lidèn and Lindblad (2011) state that the relationship between healthcare professionals, the patient and informal caregivers can be seen as a “co‐creation” and a forming of a professional friendship in a home care context.

In nursing literature, the term “co‐create” is generally understood as something beyond collaboration and different from individual creativity (Gaydos, 2005). Whereas Gaydos (2005) focused on co‐creation of narratives between the nurse and the patient, other authors emphasize the importance of co‐creation of possibilities through a process of sharing knowledge in dialogue (Andershed, 2013; Bergdahl et al., 2013; Palumbo, 2016). The results presented by Bergdahl et al. (2013) suggest that the co‐creative process could improve the dying patients’, the relatives’ and the families’ possibilities to reach vital goals in palliative home care.

In a systematic literature review on the subject of co‐production of health care and co‐creating partnerships between healthcare professionals and patients, co‐creation is described as important for the patient and for the quality of health care (Palumbo, 2016). According to McCormack and McCance (2010), person‐centred nursing requires “therapeutic relationships between professionals, patients and other important persons in their lives and these relationships are built on mutual trust.”

Several authors mention co‐creation in palliative care, for example co‐creation “… may cause vital goals to be met” in an action‐oriented process (Bergdahl et al., 2013). Planning and implementation of care should be done in a co‐creative manner (Ternestedt & Andershed, 2013), and co‐creative care is also mentioned as the best way to support hope (Benzein, 2013).

Action‐, process‐ and goal‐oriented forms of thinking are not new in nursing. Carper (1975, p. 68) stated that “Nursing is conceptualised as a deliberate, goal‐directed, action‐oriented process, which cannot be defined apart from the recipient of nursing care.” However, it is currently not known how a goal‐ and action‐oriented co‐creative process works over time in palliative home care. We believe that a theory needs to be created to give knowledge about the co‐creative process. Such a theory could be a foundation for further research and education. Hence, the aim of this study was to test the theoretical conceptualization of the co‐creative process in home care nursing encounters (HCNEs) over time in the context of advanced palliative care.

### 1.2 The conceptualization of the co‐creative process

This conceptualization of the co‐creative process is influenced by Nordenfelt's (1995) action‐oriented approach and describes how nurses, patients and relatives together can create possibilities to reach vital goals during the last journey of life (Bergdahl, et al., 2013). Vital goals are life activities that become important for patients and relatives while in palliative care, for example regaining an ability, such as going out shopping or travelling to see relatives.

**Figure 1:**
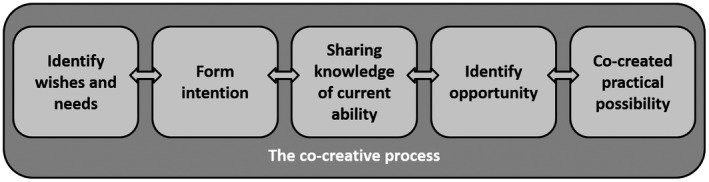
The co‐creative process

Co‐creation of practical possibilities is achieved through a five‐step process (Figure 1) consisting of “identification of wishes and needs,” “forming intention,” “sharing information on current ability,” “identification of opportunity” and finally “co‐created possibility.” For this process to take place, underlying conditions are required, such as trust and a good caring relationship. A co‐created possibility is in this context understood as achieving a vital goal related to well‐being.

## 2 METHOD

### 2.1 Design

This study was designed to test the conceptualization of the co‐creative process using a hypothetical, deductive approach. By testing the conceptualization of the co‐creative process with a deductive approach, there is a chance of finding weaknesses in the theoretical construction and thereby developing our knowledge. The power of deductive approaches lies in how they let us know which part of a conceptualization or theory that does not constitute a good explanation of the phenomenon of interest. Deductive approaches also enable researchers to find other explanations that are supported by the empirical data. The deductive approach is also a way to avoid the form of bias associated with inductive qualitative approaches. (c.f. Bergdahl & Berterö, 2015; Elo & Kyngäs, 2008).

### 2.2 Hypothesis

Two hypotheses were created based on deductive reasoning on the conceptualization of the co‐creative process:

- All five steps in the “co‐creative process” are worked on in the process, and one can go back and forward between adjacent steps.
- The amount of time spent in each home care nursing encounter is important for the co‐created process.

The hypotheses gave expectations of finding data or scenes that could be associated with each step of the co‐creative process. Not finding such scenes and not being able to follow the process would falsify the hypotheses. The hypotheses also have a sensitizing effect, making the researchers sensitive to data that have the potential to falsify the hypotheses.

### 2.3 Setting

Data was collected at two hospital‐based specialist home care units. The units were providing care to patients in a late palliative phase as well as patients in earlier phases of terminal illness and patients with long‐term illness. Both units were giving 24‐hr service in the patients’ homes supplied by a multiprofessional care team with access to inpatient care in a hospital ward. At the time of the study, most the patients, 40–50 per unit out of 60, were receiving advanced palliative home care. Both units had similar staffing, about 30 Registered Nurses (RNs), three and four physicians, respectively, a physiotherapist, a medical social worker and an occupational therapist, as well as access to a chaplain and a dietician.

### 2.4 Participants

We began by recruiting nurses to be included in the study, and each of them later suggested a patient to be included in the study. All nurses in the above‐mentioned units were invited to participate. The invitation and information were put forward by their managers both verbally and in writing. The inclusion criteria for the nurses were that they should have at least one year of experience in palliative care and that they had some further education–for a specialization–after completing their nursing degree. Three nurses agreed to participate. The nurses were between 34 and 56 years of age and had 1–15 years’ work experience in advanced palliative home care.

The participating nurses approached patients and relatives, who they and the researchers considered to be suitable for inclusion in the study. The inclusion criteria for the patients were as follows: no cognitive impairment and that they could be followed up for at least seven weeks. The nurses approached one patient each and explained the overall aim and design of the study, and all the approached patients volunteered to participate. Those patients were between 60 and 76 years old, and all had cancer in an advanced stage with metastases and had been undergoing palliative care for between 1 month and 2.5 years. During the study, none of the patients were bedridden and they led a relatively active life despite the need for regular care. Two patients had recently received palliative chemotherapy, and one of them was in ongoing chemotherapy treatment. Two of the patients had a Port‐A‐Cath and needed blood transfusions and/or parenteral nutrition. The three participating patients also had other diagnoses, such as kidney failure, heart failure and diabetes. Two of the patients were married or cohabiting with a partner that was also included in the study. Three nurses, three patients and two relatives are included in the three cases reported on in this paper.

### 2.5 Ethical consideration

In designing the study, the patients’ and the relatives’ potential vulnerability and dependency have been considered. Permission was received from the management at the advanced home care units before we initiated the study. All the participating nurses, patients and relatives received oral and written information about the study and about the possibility to withdraw from the study at any time without stating a reason. All participants signed an informed consent. To ensure confidentiality, some identifying characteristics that are not crucial for understanding the results have been changed in this paper (c.f. Kaiser, 2009). Ethical approval was received from the Regional Ethical Review Board in Stockholm, Sweden (No; 2009/916‐31).

### 2.6 Data collection

#### 2.6.1 Observations

Home care nursing encounters (HCNEs) were observed between December 2009 and September 2010. In this study, 17 observed planned HCNEs were analysed, in three cases, Case A—5 HCNE, Case B—6 HCNE and Case C—6 HCNE.

The observations started when the nurse entered the patients’ home and ended when she or he left. Each observation/encounter lasted between 15‐90 min. For increased reliability, two researchers observed each encounter (c.f. Yin, 2009). The first author (EB) participated in all observations to ensure continuity, and the other researchers took turns at participating in the HCNEs. The researchers were present in the same room as the nurse and patient during the whole observation, and in some encounters, the relative was also present in the room. The researchers noted activities performed by the nurse, the patient and the relative. The researchers also noted the verbal and non‐verbal communication and the overall atmosphere in the encounter. To perform the analysis, all notes were transcribed. Database computer software was used in the analysis process.

#### 2.6.2 Interviews

Short interviews with the nurses took place immediately before and directly after each encounter. Interviews with patients and relatives were conducted after each encounter. The interviews with the nurse before each encounter were intended to cover what the nurse planned to do in the upcoming encounter. The interviews after the encounters were intended to grasp the patients’, relatives’ and nurses’ impressions of what happened in the HCNE. The interviews with patients and relatives took place in the patient's home.

There were 33 interviews conducted with the nurses (once—no interview after) and 16 interviews with patients and four with relatives. In total, there were 53 interviews. On one occasion, the patient and their relative were interviewed together. The interviews lasted between 5‐35 min and were performed by the researchers doing the observations; one interviewed the nurse when she had left the patient's home; and the other one stayed in the patient's home and interviewed the patient and relative. The shortest interviews occurred when the nurse was in a hurry because of other obligations. All interviews were recorded and transcribed verbatim.

### 2.7 Data analysis

The data used in this analysis were observations and interviews. The observations were previously analysed (Bergdahl, et al., 2013), but in this study, the analysis was conducted in a new way, consider cases, in chronological order over time. The observations were analysed together with the interviews belonging to each case and encounter. The interviews have not previously been analysed. The analysis of the observational data was a secondary analysis; however, the primary and secondary analyses were carried out by the same researchers that were also involved in the data collection.

The aim of the analysis was to test the “co‐creative process” (Bergdahl, et al., 2013). This was done by performing a hypothetical‐deductive analysis of qualitative data (Bergdahl & Berterö, 2015; Elo & Kyngäs, 2008).

The analytical techniques methods used were pattern matching and cross‐case synthesis (Yin, 2009). Pattern matching was done by using the five steps in the co‐creative process deductively and matching them with observation and interview statements. Several data displays were constructed during the process to explain and interpret the co‐creative process. Each display was analysed and discussed regarding explanatory strength. We also analysed how well the data supported the data display. This analysis went through several iterations where we critically examined weaknesses and possible lack of fit with the data in each iteration.

Creating several versions of data displays in a trial‐and‐error manner gradually gave a deeper understanding of the complexity of the co‐creative process.

Cross‐case synthesis concluded the analysis. The intention was to understand the overall findings from all the cases and to reveal similarities and differences between the cases. Each case was first analysed separately and then in relation to the other cases (c.f. Yin, 2009).

## 3 RESULT

The overall result was that the co‐creative process was more complex than the theoretical conceptualization we started with. The five steps, “Identify wishes and needs,” “Form intention,” “Share knowledge of current ability,” “Identify opportunity” and “Co‐create practical possibility” were corroborated as usable high‐level conceptualization of the process. However, the relation between the steps was more complex, especially in relation to time.

The major findings are as follows:

- All cases contained a *main process* that was related to a vital goal for the patient.
- There were *sub‐processes* related to the main process. The subprocesses were severe symptoms or problems that were complex to treat and needed to be treated over several encounters.
- There were *micro‐processes*, simpler symptoms, side effects or problems that could be treated in one or two encounters and that were not severe.
- When some vital goal was realized, it was considered a *co‐created possibility*. Most possibilities needed to be *maintained*.
- *Time* was more important than previously understood. The main aspect with regards to time was that a long period of time with careful monitoring of progress and sensitivity to the pace of patients and relatives was needed. The findings indicate that complexity, existential implications and relatives’ involvement increase the number of encounters that are needed to achieve co‐creation.

To illustrate the result and the analytical process, we present five encounters from one of the cases (Case A). The reason we choose this case to illustrate the result is that it is a pedagogic case that clearly illustrates important aspects of the co‐creative process. The boxes link the events in the encounters to the findings and the five steps of the theory.

### 3.1 Case A—Regain appetite and be free of the PN and Relieve relative's anxiety

This patient was 65 years old and lived with her husband. She had worked as a secretary but was now retired. She had spreading gynaecologic cancer that had been diagnosed one year before she was admitted to advanced palliative home care. She recently had surgery and chemotherapy. Because of this, she had lost a lot of weight and now her weight was only 47 kg and she needed parenteral nutrition (PN).

In the interview before the first observed HCNE, the nurse gave us some additional information. It was important for this couple to eat good food and enjoy a glass of wine, and the husband enjoyed cooking for his wife. He was also very worried about his wife's health, and the nurse was aware of his anxiety. The patient wanted to be free from the PN and regain her appetite; this was a vital goal for her*.* The intention in the upcoming encounter was to flush the Port‐A‐Cath system and disconnect the PN drip.

#### 3.1.1 An atmosphere of friendship: The first observed encounter

The nurse and patient discussed nutritional supplements, while the nurse performed care tasks. The patient wanted to get her appetite for food back. Together, they planned nutrition and decided to go without the PN during the upcoming week in the hope that it would stimulate the patient's appetite. They also planned how much supplement drink the patient needed.

See Box 1 for theoretical linkages.

Box 1 1 The planning of the PN and supplement drinks is an *identified opportunity* to reach an important vital goal for the patient, to be free of the PN and regain appetite. This turned out to be the main process.

During the HCNE, the husband seemed anxious and asked a lot of questions. He wanted medical facts about his wife's health. When the nurse was ready to leave, the husband followed her to the door and started a discussion. The nurse focused on the husband. She told him that, if they could reduce the use of PN, it might stimulate his wife's appetite. The husband asked the nurse if he should “pressure” his wife to eat more. The nurse advised against this. She reassured him that they could make a new decision regarding the PN later.

See Box 2 for theoretical linkages.

Box 2 1 The nurse *identified* the husband's *need* and gave him time to share his concern. The anxiety of the husband was triggered by the patient's *wish* and plans, to be free of the PN. By *sharing knowledge about the patient's current ability* and *situation,* the nurse could increase the husband's knowledge so that he would *re‐form his intention* to pressure his wife to eat more. In this way, the main processes of *Regain appetite and be free of the PN* and *Relieve relative's anxiety* were interdependent and the relative's anxiety was a closely related sub‐process.

In the interview following the HCNE, the nurse spoke about her intended plan to work with the patient to try to reduce the PN and that she had intentionally encouraged the husband to ask questions. She recognized that he needed support from her, and she regarded it as an important part of the care:

We often have this ‘meeting’ at the front door when I'm about to leave. He wants everything in writing, he needs facts and figures; otherwise he becomes more anxious.

#### 3.1.2 A balancing act: Encounter two

In the interview before the encounter, the nurse said that she wanted to help the patient to continue with her plan to be free of the PN. The nurse regarded the care as a balancing act. She let the patient decide for herself regarding the PN and tried not to pressure her while at the same time considering the patient's low weight and other possible problems that could occur.

At the beginning of the encounter, the patient said that she had weighed herself. The nurse noted the weight and took her blood pressure. She then asked how the patient felt now when she had been without the PN for a week. The patient answered, with joy in her voice, that she felt much healthier without the PN.

See Box 3 for theoretical linkages.

Box 3 1 An *opportunity* or *partial possibility* on the way to the goal of being free from PN was realized. The *wish* regarding a regained appetite remained to be realized. It is important to notice that the patient, even at this early stage of the process, felt much healthier.

The patient and nurse discussed nutritional supplements again, and the nurse confirmed that the patient was taking the correct dosage. The patient was worried because she was not gaining weight. The nurse calmed the patient and told her that it was normal and that it would take some time to gain weight. During the conversation, they held each other's hands and made eye contact. The nurse's body language showed that she cared about the patient.

#### 3.1.3 A caring disclosure: Encounter three

In this encounter, the physician on the unit accompanied the nurse. The intention of the physician was to get updated and to obtain information about the patient's recent visit to the oncologist.

When the nurse met the patient, she handed over four nutritional drinks. The patient commented that it was the flavour she liked most. The husband looked worried and asked questions about the lack of appetite that the patient was still experiencing.

The husband now revealed that the patient was taking nicotine gum. He said that he suspected that it was affecting her appetite negatively and that he wanted her to stop taking it.

The doctor confirmed that he might be right about the nicotine gum. The patient looked uncomfortable with the situation. The physician said that it was a good thing to have this open conversation.

See Box 4 for theoretical linkages.

Box 4 1 The husband cared for and was anxious about his wife and tried to *re‐form his wife's intention*. He revealed and *shared knowledge* about her habit of chewing nicotine gum.

The nurse said in the interview that this encounter highlighted the special relationship this couple had.

The husband said in his interview:

My wife doesn’t want me to talk about this, I know that. But I feel it is important that facts are presented. And it isn’t good for her to continue with this gum; it affects her appetite and I wanted the physician to know about it and I wanted to hear her view on it as well and she said that it’s easier to gain weight without nicotine gum.

See Box 5 for theoretical linkages.

Box 5 1 The husband confirmed that he had intended to *re‐form the intention* of his wife.

#### 3.1.4 The best diet cure available: Encounter 4

Before the encounter, the nurse said that she intended to get an update on the patient's situation and to monitor glucose and blood values since the patient was no longer receiving the PN.

The patient mentioned a tingling sensation in her fingers that she had recently started to experience. The nurse noted this and promised to investigate it further.

In the encounter, the nurse asked the patient how her appetite was coming along. The patient answered: “*My situation is the best diet cure available*,” and laughed.

While preparing the treatments, the nurse asked the patient if she was not missing the PN. The patient answered that she was not.

See Box 6 for theoretical linkages.

Box 6 1 *Knowledge* was being *shared* while nursing acts were performed. The nurse *re‐identified* the patient's *wish* to be without the PN.

The nurse also *identified* the issue with tingling in the fingers.

They walked towards the door, and the nurse asked the patient how her husband was coping with the situation. The husband was asleep in another room. The nurse and patient had an intimate discussion about the husband. The nurse promised to mention his anxiety to the physician. They made a plan that the husband should come to the physician's office to discuss the situation and his concerns with both the physician and the nurse.

See Box 7 for theoretical linkages.

Box 7 1 The nurse reacted to the patient's information regarding the *relative's anxiety,* and they co‐created an *opportunity* for the husband to discuss the situation.

In the interview following the HCNE, the nurse said:

She [the patient] is a bit worried about the husband's anxiety, so I offered to arrange for him to have a meeting with me to discuss his experiences

The patient told the interviewer that she felt safe and was able to bring up anything with the nurse:

I never feel stress in these meetings, so I can always bring up any subject, even existential issues. But there has not been any reason to discuss such things and they always ask me how I feel. I feel relaxed when they come. It is wonderful to be able to get this care in your own home. The nurse always thinks one step ahead and offers solutions.

#### 3.1.5 A regained appetite: Encounter five

The nurse planned to administer medication, flush the Port‐A‐Cath system and take blood samples. According to the nurse, the patient had received cortisone and felt a lot more alert, had a better appetite and did not need the PN. The husband had now had a private discussion with the nurse and the physician. This had reassured him about the treatment, and he seemed less anxious. The nurse also explained that she had investigated the issue of tingling in the fingers; it was a side effect of the cytostatic drugs and a B‐vitamin supplement should help. The patient said that it was a relief to know this and that the problem was not so severe at that time.

See Box 8 for theoretical linkages.

Box 8 1 A co‐created practical possibility was reached for the goal of Regain appetite and be free of the PN; also, the relative's anxiety had been relieved.

The issue with tingling was resolved. It was considered a *microprocess*.

At the beginning of the HCNE, the nurse rested her hands on her knees and leaned forward towards the patient and asked how she felt. The patient replied that she felt a lot better, almost as well as before she became ill. She even had the energy to take several short walks. The patient started to describe a stroll she had recently taken in a park. She went on and said that she wished that she could gain some more weight. The nurse did not comment verbally; she just nodded. The nurse performed the care tasks while the patient talked.

When the nurse was ready to leave, the husband followed her to the front door again. He said that he was worried about his wife's not gaining weight. The nurse explained that gaining weight could take some time and the lack of gained weight could be due to the patient being more physically active, which in itself was very positive.

In the interview following the HCNE, the nurse explained that she was glad that the patient was feeling better. Now, there was nothing serious troubling the patient, but the nurse was still concerned about the weight. She intended to keep track of the patient's weight and to work on her nutrition to make the patient stronger. The nurse was also careful not to worry the patient:

You can’t rush things, they must not feel pressured. That’s the reason why I avoided asking about the patient’s weight, it might take some time but she is feeling better now. I really hope she’ll gain weight and get stronger. We need to monitor her state so that she can feel this way as long as possible.

When asked to reflect on the caring relationship, the nurse answered:

I feel that the relationship with the patient and her husband gets deeper and stronger each time we meet and I get to know them better, their background where they grew up and such things. It’s interesting.”

In the interview, the patient confirmed that she was feeling good:

Food tastes good now. It’s wonderful! It tastes good again and I even enjoy a glass of wine! I have always enjoyed a good wine with the food and then, suddenly, it didn’t taste like anything and I was so disappointed. But now it tastes good again, it’s a joy.

### 3.2 Conclusion drawn from the case

There was one *main process* that was aimed at reaching the patient's vital goal of regaining appetite. There was also an intimately linked *sub‐process*, the relative's anxiety. *Sub‐processes* are processes that are strongly related to the main process but are of lesser importance with regard to the vital goals. There was a wish to be able to handle the stoma and a sensation of abdominal pain and heaviness that are also regarded as *sub‐processes*. These issues were not resolved during our observations but were handled in such a way that they did not disturb the focus on resolving the main process. There were also two problems that were quickly resolved that were connected to side effects of the medication. The two problems, conceptualized as *microprocesses*, were a tingling sensation in the fingers, mentioned above, and some perceived hearing loss. These issues were resolved by the nurse. She gave information to the patient and the relative.

**Figure 2:**
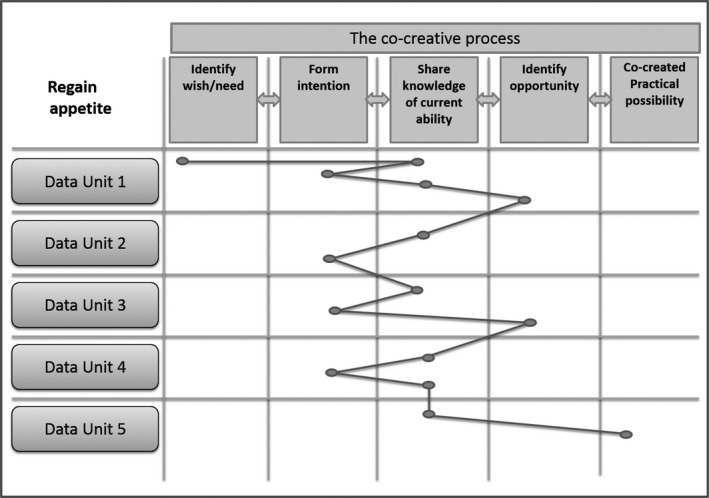
Data display of process "regain appetite" overtime, each data unit represents a HCNE

In this case, three of the processes came to be co‐created possibilities for the patient: *Regain appetite and be free of the PN*,* Relieve relative's anxiety* and *Unusual tingling bodily sensations*. The two more complex processes needed to be continuously worked on to be sustained as long as possible. The simpler processes did not need further work. Since the context is end of life care, the co‐created possibilities are of course limited in time. They can be seen as a temporary regaining of some ability the patient thought was lost and were therefore vital for the patient's quality of life. In this particular case, the main process involved a risk of losing weight which was existentially loaded, especially for the husband. The risk of weight loss also constituted a medical risk which all involved parties were aware of. The medical aspect and the existential aspect were closely related. Figure 2, shows how the main process moved over time between the three middle steps in the co‐creative process. It shows that complex, co‐creative problem‐solving is not a linear process.

We proceeded to create displays for all processes in “Case A.” This created a pattern: The three “middle” steps of the co‐creative process were “revisited” repeatedly and are conceptualized as a shape and reshape movement within each process in a manner that was much more complex than anticipated.

**Figure 3:**
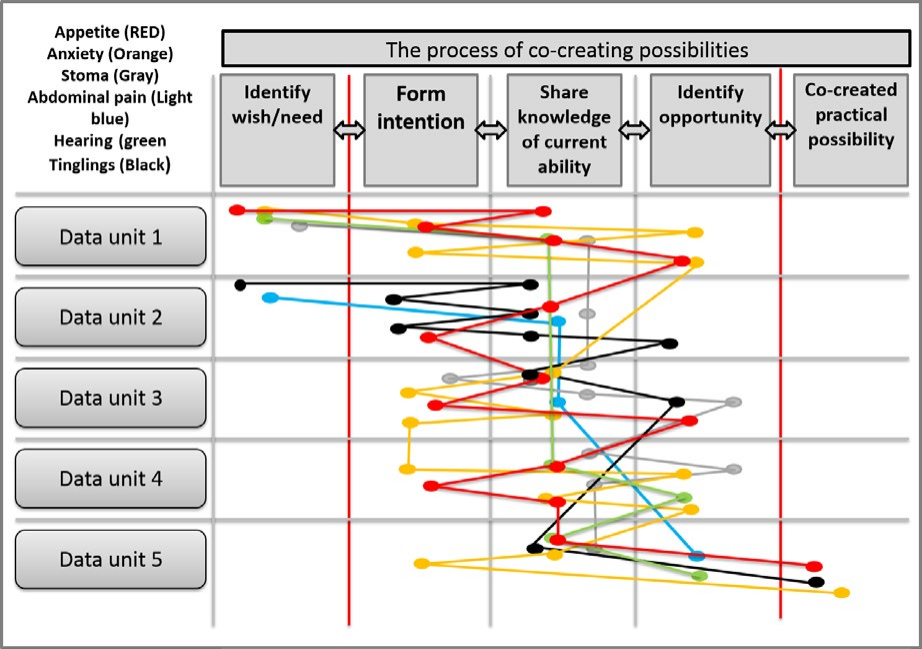
Overview of all the processes in case A, each data unit represents a HCNE

When all processes in the case are considered, the overall complexity of palliative home care encounters is illustrated. Figure 3 is an overview of all the processes in Case A. It also presents the reconceptualization of the steps in the co‐creative process that was a result of the deductive analysis. In the co‐creative process, the three middle steps were revisited several times in each process. These steps can be regarded as a *working process*, a shaping and reshaping movement, within the co‐created process.

All the processes were worked on over time, but there were different sub‐processes in focus at different points in time. The main process was always in focus, but in some HCNEs, other problems were more actively discussed or worked on.

The main process in Case A took time, due to the complexity and the closely related process of the husband's anxiety. Several weeks were needed to get him comfortable with the process. There was also a need for the nurse to adjust her approach to the changing emotional reaction from both the husband and the patient. The nurse had to balance the patient's will to be free of the PN, the relative's anxiety and the existential issues. Rushing could have jeopardized the whole process. This is an example of the mutual dependency that sometimes exists between the main and sub‐processes. The nurse supported the co‐creation process by adapting to the pace of the patient and the relative. In this way, the aspect of time needed to reach a goal is connected to the complexity of the main process and connected sub‐processes. However, the length of the home care encounters was of lesser importance, which was a surprise to us.

**Figure 4:**
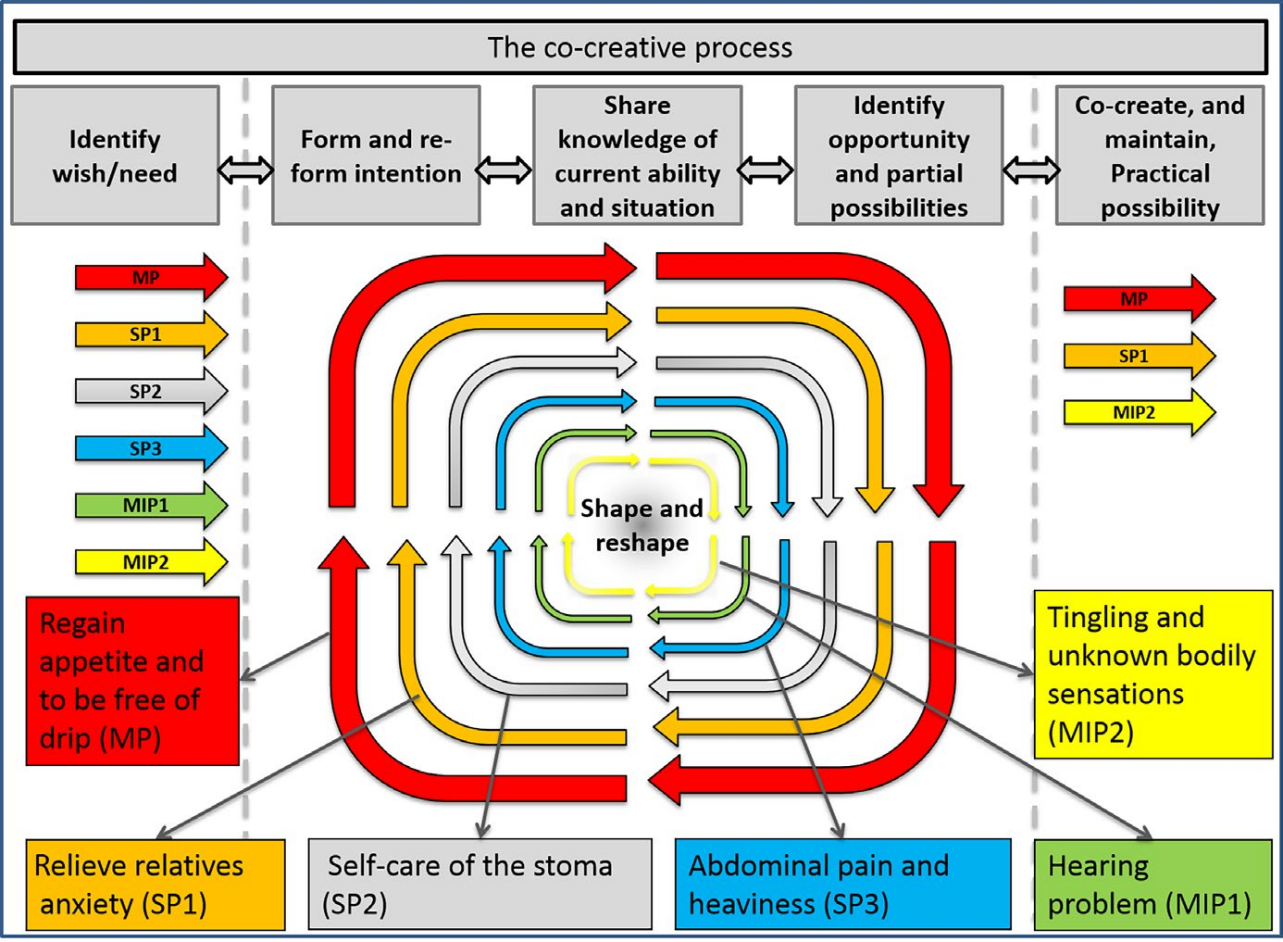
The co‐creative process in case A. The main process is marked as (MP), sub‐processes as (SP) and the micro‐processes as (MIP)

The interpretation of the time aspect was also prominent in the other cases. With this understanding, the theory was further developed and presented in the model below, see Figure 4.

The model in Figure 4 represents Case A, and the main process is the largest process in the model. The main process is marked as (MP), sub‐processes as (SP) and the microprocesses as (MIP). The model clarifies the shape and reshape characters of steps two, three and four of the co‐creative process.

### 3.3 The results of the cross‐case synthesis

In the cross‐case synthesis, the main, sub‐ and microprocesses were a useful conceptualization that functioned as a description for all cases. There was also a shaping and reshaping movement in all the cases. The main process in Case B was “Control pain so that patient can take daily walks.” Case B involved a relative who displayed some anxiety, but not at the same level as in Case A. In Case C, the main process was “Handle fear of needles, despite need for daily injections.”.

There were also linked sub‐processes, which had a medical connection to the main processes in all cases. In addition, there were “unlinked microprocesses” which were issues that needed resolving but were not directly linked to the main process. The microprocesses were less important processes that needed to be addressed to direct attention back towards the main process and the patient's vital goals.

In Case B, a sub‐process that was existentially charged was found in a “Wish for further chemotherapy.” The patient viewed chemotherapy as a lifeline; if he was receiving treatment, there was hope of keeping death at bay. In a discussion about the treatment, the nurse in Case B once tried to initiate an existential conversation connected to the possibility that the chemotherapy would end. But the patient and his wife made it clear that they did not want to have this conversation and the nurse respected their wish.

The patient in Case C was living alone. The relationship between the nurse and the patient was closer and more familiar than in the other cases. The main process was connected to daily fragmin injections that were problematic due to the patient's fear of needles. The solution to the problem was that the patient would learn to give herself the injections, despite the fear. The main goal was to increase the independence and regain more freedom despite severe illness.

The nurse trained the patient over several HCNEs and increased her courage and knowledge, which led to a co‐created possibility of a goal that also was maintained over time. Another important process concerned a weekend cruise. At first, the patient mentioned the trip to the nurse but added that it would probably not be possible due to the injections needed. The nurse objected and said that the patient should go. At the next encounter, the nurse brought up the weekend cruise and said that he had spoken to the nurse on board the ship, who had agreed to administer the injections during the cruise. With this concern removed, the cruise became a practical possibility. What surprised us in this case was that the HCNEs were briefer than in the other cases and that a complex problem was resolved in just a few weeks. Our hypothesis that the amount of time spent in the HCNE would be important for the co‐created process could not be supported.

In Case B, the following microprocesses became co‐created possibilities: “Control pain so that patient can take daily walks,” “Handle difficulty to swallow,” “Wish for further chemotherapy” and “Constipation,” and all of them needed to be maintained over a period of time. In Case C, all microprocesses became co‐created possibilities and all but “Going on a short cruise” needed to be maintained.

It was the main processes that received the most attention and were most worked on in the HCNEs in all cases. Some processes were worked on over a longer time, and some were resolved in one or two encounters. We did not find clear support regarding the hypothesis about the importance of time in each encounter. The length of encounters does not seem to have a clear importance; instead, we found that the number of encounters over time was important. The pace of the process was set in a co‐creative manner where the nurses presented or confirmed that there were possibilities to reach vital goals, and then the nurses let the idea of a possibility mature in the patients’ and relatives’ minds. This waiting is part of the “forming intention” process step; knowledge sharing over time led to a co‐identified opportunity. The shape and reshape movement between the three middle steps of the process set the pace. Forming intention is supported by sharing knowledge, and opportunities are re‐identified until a practical possibility is co‐created. The hypothesis regarding the steps of the process was expanded and altered due to the findings. Practical possibilities were co‐created that enabled the patients and relatives to reach vital goals. These possibilities were created over time, over several HCNEs, in a complex process involving several sub‐processes. Table 1 presents the main, sub‐ or microprocesses for Cases A, B and C.

**Table 1:** The main, sub‐ or micro‐processes for Cases A, B and C

Case A	Case B	Case C
Regain appetite and be free of the PN *Main‐process*	Control pain so that patient can take daily walks *Main‐process*	Handle fear of needles, despite need for daily injections *Closely related sub‐process*
Relieve relative's anxiety *Closely related sub‐process*	Gain and keep weight *Micro‐process*	Be able to take daily walks *Micro‐process*
Self‐care of the stoma *Related micro‐process*	Handle difficulty in swallowing *Micro‐process*	Go on a short cruise *Main ‐process*
Abdominal pain and heaviness *Related micro‐process*	Unknown tingling bodily sensations *Micro‐process*	Unknown tingling bodily sensations *Micro‐process*
Hearing problem *Micro‐process*	Support the relative *Closely related sub‐process*	Regain appetite *Micro‐process*
Unknown tingling bodily sensations *Micro‐process*	Wish for further chemotherapy *Closely related sub‐process*	Be prepared for and able to handle, abdominal pains *Closely related sub‐process*
	Constipation *Micro‐process*	

In the interviews, all nurses stated that there was sometimes a high workload and they tried to work in such a manner that patients would not notice the stress. One nurse discussed how she handled the workload:

We have a heavy workload now but the patient should not suffer because of that. I work late on the administration and such. I don’t want to rush and make things worse for the patients, I’m there for them. It’s very important not to rush things.

However, in some encounters, the nurse clearly stated that there had been a stressful day and a lack of time and the HCNE was kept short. When the nurse was open with regard to the workload, some complex problems were solved efficiently and with cooperation from patient and relatives and there were no negative reactions. This is an interesting aspect of co‐creation; patients and relatives in many ways want to help the nurse.

All patients said that they trusted the nurse and felt free to bring up any topic, even existential issues, such as death and dying. However, they all added that they had not yet felt a need for such a discussion at this stage. One patient put it like this:

I feel safe with the nurse, we can talk about anything and it feels good. Even existential things – but I have not felt that I wanted such a discussion yet.

The nurses said that these patients did not need to talk about death and dying at that moment, and if they brought up the topic, it could harm the relationship. As one nurse put it:

Hope is important for them in this situation. I can’t go in and encourage the couple to prepare for the patient’s death. It would destroy our relationship. They have a right to live for the day and we make the best of the situation. I try to follow them and handle the symptoms, problems and issues they have at that time.

The patients reported that they felt a trust that would make it possible to have an existential conversation but that they had not yet felt the need to. The nurses all stated that they would not initiate such a discussion unless they felt that the patients had a need for it. We also found that the processes often had existential implications and that existential anxiety was important for the pace of the co‐creative process.

### 3.4 Concluding remarks on the findings

The co‐creative process in HCNEs is far more complex and complicated than previously research has shown (Bergdahl, et al., 2013). The analysis led to a new understanding of how the nurse, patient and relative acted and talked to create possibilities. There were several parallel processes, several symptoms, wishes, problems and needs that were worked on in each encounter. Most were connected to specific vital goals that the patient and the relatives wanted to achieve over time. In all the cases, there were *main processes*,* sub‐processes* and *microprocesses*. The *main processes* all concerned needs and wishes of the highest priority for the patient, the vital goals. *Sub‐processes* were linked to the main processes, both in a medical and in an existential way. Microprocesses were symptoms that popped up and could be treated or handled in a few encounters.

**Figure 5:**
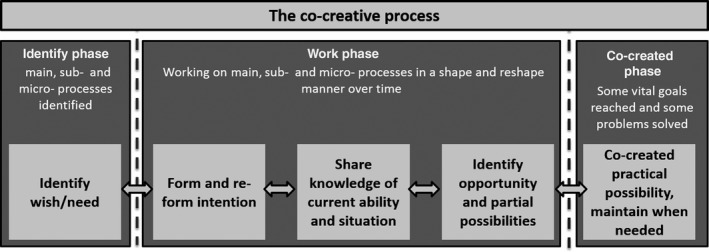
The co‐created process as result of this study

In the cases we followed, pain management was a means to an end, not an end in itself. The main processes comprised the things the patient was able to do when pain was under control. There were several occasions where the patients accepted some pain and discomfort to be able to reach vital goals. There are examples in all cases. In Case A, the patient risked discomfort to regain appetite; in Case B, the patient risked acute pain when going on short walks; and in Case C, the patient risked inconvenience and faced her fear of needles to go on a cruise. The five stages of the co‐created process are all found in the main, sub‐ and microprocesses. However, the three process steps in the middle were more clearly understood and seen as a shape and reshape working phase in the overall process. Thus, the co‐creating process can be said to have three phases: an identification phase, a working phase and a phase where a co‐created possibility was reached. Our understanding on a co‐created possibility was also deepened. It was clear that the ability that constituted the co‐created possibility needed to be maintained for the most part. The deepened understanding of the co‐created process is presented in Figure 5.

The model and conceptualization of main, sub‐ and microprocesses in the co‐creative process and the importance of time constitute the further developed theory and main result of this paper. In each of the three cases, six to seven processes were found. The main, sub‐ or microprocesses are defined as parts of a caring process aimed at reaching a goal related to the patient's well‐being. In each HCNE, several processes were worked on and discussed at the same time. Many of the processes were complex and included different treatments tailored for the patient in a caring manner. The processes were dynamic, interrelated and unique for each patient.

Regarding the two hypotheses that formed the framework for this study, the first hypothesis was as follows:

- All five steps in the “co‐creative process” are worked on in the process, and one can go backward and forward between adjacent steps.

This hypothesis was refined since the back and forth movements are mainly among the three middle steps,.

The second hypothesis was as follows:

- The amount of time spent in each HCNE is important for the co‐created process

We found no clear support for this hypothesis. However, time was important but in another way. Some processes needed to be worked on in several encounters over several weeks. These were complex processes often involving an existential dimension, as described above.

## 4 DISCUSSION

The conceptualization of the complex main, sub‐ and microprocesses in the co‐creative process and the importance of time constitute the further developed theory and main result in this paper. These complex parallel processes were unexpected. This study shows that reaching vital goals involves a complex process over a period of time. In the co‐creative process, the nurses handled the “shape and reshape” movement in the HCNE using a professional perceptive and person‐centred approach to palliative care focusing on the best possible way to reach the goals vital for the well‐being of the patient and relatives.

Seeing co‐creation as a complex but understandable process is a way to conceptualize how “Home care is jointly shaped by health professionals, patients and family members …” (Lindahl et al., 2011, p. 461). Lindahl and co‐authors found that co‐created home care is based on what they describe as “professional friendship.” That type of deep personal relationship can be developed through sharing information (c.f. Barnard et al., 2006). The continuous sharing of knowledge in the home care encounters is a tool that was used to shape care to support the patients’ well‐being or “total good” (c.f. Randall & Downie, 1996). The total good is also close to the notion of vital goals. However, vital goals are more concrete, and an advantage with the model used is that it clarifies how to work towards the ideals of palliative care in manageable processes where each sub‐process has a goal related to the patient's happiness and well‐being.

Gaydos (2005) see the co‐creation as a creative, spontaneous and unpredictable process which touches on the notion of nursing as an aesthetic practice. The shaping and reshaping movement could represent a unique aesthetic pattern of a good caring relationship and how the participants in the HCNE work together towards goals for the patients and relatives’ well‐being. Being concerned for the patient's well‐being is essential for nurses in palliative care according to Randall and Downie (1996). The new understanding of the co‐creative process can be a way to illustrate and explain how “informed caring for the well‐being of others” (c.f. Swanson, 1993, p. 357) can be performed in advanced palliative home care. This novel way of conceptualizing the theory of the co‐creative process over time could be a way to develop the curriculum and to educate nurses on how to reach the goals of palliative care for their patients and families. The model can also be used as a tool in supervision for nurses in clinical practice (c.f.Bergdahl, Benzein, Ternestedt & Andershed, 2011).

In this study, we found that the nurses made sure they adjusted the pace of the process to the needs of the patients and relatives, and one could say that they were perceptive to the patients and relative's needs. Perceptiveness has been conceptualized as an important ability for expert nurses to create good caring relationships (Bergdahl, Wikström & Andershed, 2007) and as a core approach in dignity‐conserving care action in palliative care (Harstäde, Blomberg, Benzein, & Östlund, 2017). Perceptiveness was also evident in the way the nurses handled time, making sure that the patients and relatives are involved and thereby avoiding doing or saying things they are not prepared to handle. In this way, time also had an existential dimension. Giving patients and relatives time is about respect and adjusting to the patients’ needs and preparedness for giving information and having existential conversations. Several authors argue that, if existential conversations are offered, it needs to be done in a cautious and respectful manner; however, there is a fine balance between being too careful and unconsciously avoiding sensitive subjects (Milberg & Strang, 2011; Randall & Downie, 1996; Sand, Olsson, & Strang, 2009). The importance of existential issues was also connected to how much time that was needed to reach a vital goal. Complex processes, with existential implications that had an impact on the relatives, are the ones that needed most time in this study. An important aspect was also that the nurses did not rush things, for example, in Case A, regaining appetite and being free of PN took five weeks. Five weeks might seem a long time in palliative home care, but since the process was co‐created, the HCNEs were planned with the patient and relatives and there was also a continuous advancement of the patient's well‐being over time. Since these main, sub‐ and microprocesses were worked on over time in the co‐created process, it is also reasonable to assume that continuity is important for the advancement of the processes and for the caring relationship to develop, this is an issue that should be taken into consideration when planning the organization of palliative home care.

Swanson found that the nurse “… modulates care between what she or he needs to do to assure safety and what the client must do to learn self‐care” (Swanson, 1993, p. 353). An example of such modulation is the main process in Case A, “Regain appetite and be free of the PN,” the nurse acted as an expert and balanced the medical responsibility in the situation with the patient's wish and ability to manage her own nutrition to reach the goal in a caring manner. Devik, Hellzen, and Enmarker (2016) point out that nursing and palliative care often are disease‐ and symptom‐focused and therefore might miss other factors that are important to support well‐being. The nurses in our study seem to regard symptom and pain management as a means to an end, or a tool to reach vital goals for the patients, relatives and the goal of palliative care, quality of life. The nurse–patient–relative/family relationship, especially in palliative care, involves periods of interpersonal engagement and a greater focus on trust and closeness, compared with the traditional medical encounter. Alleviating severe symptoms for the patient can be seen as a way to establish trust and shift focus to create possibilities for the patient to reach, vital goals and thereby experience happiness, an important aspect of health according to Nordenfelt (1995).

The main, sub‐ and microprocesses, as part of the developed theory co‐creative process, can be understood as a concretization of how nursing can be performed within the context of palliative home care. They show how nurses need to synthesize knowledge from different areas to assist patients and relatives in reaching vital goals (c.f. James, Andershed, Gustavsson, & Ternestedt, 2010).

This theoretical model can help nurses to analyse complex care processes and could also serve as a reflective tool for explanations of how nurses can co‐create possibilities for patients to reach vital goals in end of life care.

### 4.1 Limitations, method discussion and suggestions for further inquiry

By using a hypothetical‐deductive approach in testing the conceptualization of the co‐created process, we are aiming to avoid the limitations of inductive qualitative methods. Inductive methods often settle for descriptions of phenomena and thereby lacks in ability to present explanation in the form of scientific theory. With a deductive method, some theoretical assumptions can be falsified and other assumptions are corroborated. The creation of a theory also creates possibilities for revaluation of experiences and the invention of new experiences (c.f. Feyerabend, 2010). By looking at the data with a theoretical lens, we are both testing the theoretical conceptualization and discovering new aspects of the data.

Combining observations and interviews gave us the opportunity to obtain diverse views of the phenomena studied. By using these two data collection methods, we gained increased construct validity in the study (c.f. Yin^2009^). This type of multiple data collection and analysis is common in grounded theory studies and ethnographic studies. We found one study with a very similar data collection method, by McKenzie et al. (2007). They collected data before and after an encounter. They did not, however, collect data over time. We believe that, if a care process is to be studied, the data collection should be performed over time. We used deductive analysis and this approach is not frequently used in qualitative research, but it is a method that is recommended for use in case studies (Flyvbjerg, 2001; Yin^2009^). Some of the data in this study have been analysed and published in a previous study that focused on each encounter. That study formulated the hypothesis, the co‐creative process, which was tested in this study. In one sense, we performed a secondary analysis; however, the critical deductive approach avoided bias by critically testing the previous conceptualizations. It also needs to be pointed out that all data were collected by the same researchers that conducted this analysis and contributed to this paper. In that way, most of the limitations associated with a secondary study, such as that the data were collected by other researchers and with a different aim, are not applicable to this study. In this study, we explicitly avoided verification of previous results; instead, the whole process used in writing this paper and in the secondary analysis was a way to critically test and maybe enhance previous conceptualizations and results.

Three cases were included in this study, which can be regarded as a weakness if one is influenced by quantitative inductive methods. Since this is a qualitative case study and we used both pattern matching and cross‐case analysis, we believe that the findings are a good foundation for our theoretical conclusion. We sought to describe good examples of palliative home care encounters to form a theory about how to deliver good palliative home care. In that way, the sampling of cases was in line with the aim of this study.

One weakness of the study may be that we asked the nurses to recruit the participating patients and relatives. It is reasonable to assume that they recruited patients they felt comfortable with or had a good and caring relationship with. On the other hand, it is not necessarily a disadvantage that we choose to observe caring relationships that could be said to be good since it is important to spread knowledge about practice that enhances the patient's well‐being. Benner and Gordon (1996), among others, argue that we need a better understanding of skills that are needed to reach good care to support practice. Since our approach was to deductively test a general conceptualization, not to inductively verify results or to generalize to a population, bias in the traditional, inductive sense was not considered to be a problem (c.f. Yin^2009^). The cases we examined were of the “most likely” type, which are especially well suited for falsification of a hypothesis. If the hypothesis is falsified in a most likely case, it means that the hypothesis will probably not fit any real case (c.f. Flyvbjerg, 2001). Each reader will need to use their own judgement to assess the arguments given in this paper and to determine how best to apply the results in their practice. In this, we see ourselves as connected to the pragmatic tradition in evaluating a theory based on its usability in practice as well as in further research.

The data analysed were from planned HCNEs. In future studies, we should include acute HCNEs to obtain a more comprehensive picture of advanced palliative home care. Further studies are needed which should include critical tests of the theory, again in palliative care but also in other contexts of advanced care. We believe that this theory could be used in palliative care nursing research and further research is needed to find conditions and contexts that prevent nurses and patients to reach vital goals.

## 5 CONCLUSION

The further developed theory of the co‐creative process and its main, sub‐ and microprocesses can be understood as a concretization of how good nursing care can be performed in the context of advanced palliative home care. The different processes in all cases were aimed at the patients’ well‐being and at supporting them in reaching their goal at the end of life. Our conclusion is that the co‐creation of possibilities for patients and relatives is complex and that several problems needed to be worked on to reach vital goals. It can also be concluded that such a possibility‐ and goal‐driven approach might lead to better outcomes for palliative home care and may improve the quality of life of patients and their families facing problems associated with life‐threatening illness. The purpose of this care was to use the “tools” available to achieve the possibility for the patient and relatives to reach their goal at the end of life and thereby enhance their quality of life and well‐being.

## 6 RELEVANCE TO CLINICAL PRACTICE

This study indicates that nurses should co‐create possibilities for patients and their relatives to reach vital goals, and this means that nurses in practice should attempt to have dialogues with the patient and relatives, to identify which achievable vital goals they have and then co‐create possibilities. In this way, the medical aspects of the care are seen as means that can be used to reach vital goals and thereby achieving person‐centred care.

The model can be used in clinical practice as a reflective tool. The theory with its different steps can help nurses to handle the complex reality of palliative home care encounters and co‐create possibilities for patients to reach vital goals in end of life care.
